# A Common Polymorphism of Upstream Transcription Factor 1 Gene is associated with Lipid Profile: A Study in Chinese Type 2 Diabetes Families

**Published:** 2009-09

**Authors:** Yan Song, Na Li, Liu He, Tang Xun, Dafang Chen, Yonghua Hu

**Affiliations:** *Department of Epidemiology and Biostatistics, School of Public Health, Peking University, Beijing, China; Key Laboratory of Epidemiology, Ministry of Education, Beijing, China*

**Keywords:** dyslipidemia, genetic epidemiology, single nucleotide polymorphism, type 2 diabetes mellitus, upstream transcription factor 1

## Abstract

Upstream transcription factor 1 (USF1) is capable of controlling various members in glucose and lipid metabolism pathways. Much evidence suggests that the rs3737787 polymorphism in the USF1 gene may lead to alteration of lipid metabolism. The objective of this study was to test the association between rs3737787 and type 2 diabetes mellitus (T2DM) and its related lipid metabolic traits in the Chinese population. A total of 287 eligible T2DM families were chosen in Beijing. A set of questionnaires was administered to obtain information on demographic characteristics. Physical measurements were recorded. DNA was extracted from blood samples and genotyped using PCR-RFLP method. Statistical analyses including linkage analysis and family-based association test were performed to detect the relationship between rs3737787 and T2DM related traits. In the non-parametric linkage analysis, it was observed that rs3737787 is potentially linked with triglyceride and apolipoprotein E levels, where the logarithm of the odds scores were 0.87 (*p*=0.02) and 1.96 (*p*=0.001), respectively. Similar results were obtained in the multi-factorial generalized estimating equation analysis. Using different statistical approaches, in this study, we have confirmed that the single nucleotide polymorphism rs3737787 is related to triglyceride and apolipoprotein E levels in type 2 diabetes mellitus families.

## INTRODUCTION

Dyslipidemia is the main cardiovascular risk factor of type 2 diabetes mellitus (T2DM) patients, and may potentially lead to some severe complications, including many common cardiovascular and cerebrovascular diseases. Lipid metabolism of T2DM is modulated by several factors, in which control of blood glucose and insulin resistance are the most important, while insulin resistance is the central link resulting dyslipidemia. Insulin resistance results in increased serum concentrations of very low density lipoprotein (VLDL) and triglyceride (TG) and decreased rates of their clearance. It was found that dyslipidemia is a major modifiable risk factor for atherosclerosis (1), which introduced the possibilities of either treatment or prevention of the complications of T2DM. Therefore, it is necessary to study the relationship between candidate genes of T2DM and dyslipidemia of T2DM patients so as to perform different and appropriate prevention and treatment on these individuals according to their different risk level studied from genotyping of the candidate genes.

Upstream transcription factor 1 (USF1) is a ubiquitously expressed transcription factor, and is a member of the basic helix-loop-helix leucine zipper family. USF1 has been shown to modulate the expression of various crucial genes related to glucose and lipid metabolism, such as glucokinase, fatty acid synthase, and acetyl-CoA carboxylase gene, by specifically binding to E-box motifs in these genes. The important role of USF1 in regulating and coordinating various members in glucose and lipid metabolism pathways leads it to be regarded as a significant candidate gene for some metabolic abnormalities such as T2DM, dyslipidemia, and metabolic syndrome. *USF1* gene is located at human chromosome 1q22-q23, which is the most consistently replicated locus showing linkage with T2DM in different populations (2–10).

*USF1* was initially reported to be linked and associated with familial combined hyperlipidemia (FCHL) in Finnish families and in other populations (11). As FCHL and T2DM have similar characteristics, *USF1* was also thought to be an important susceptibility gene of T2DM and its related traits, especially dyslipidemia. The research conducted by Ayyobi *et al* (12) indicates that patients with FCHL show evidence of insulin resistance as well. This suggests that obesity and insulin also play vital roles in the FCHL-related lipid metabolism disorders, and maybe the etiological foundation of FCHL. Therefore, some researchers believe there is some overlap in the etiological foundation of the two diseases, but the extent of the overlap is difficult to assess since the detailed mechanisms of FCHL and T2DM have not been elucidated (13).

The most commonly studied single nucleotide polymorphisms (SNPs) of *USF1* gene are rs3737787, rs2073653, rs2073655, rs2073658, rs251640, rs2516841, rs2516839, rs2774276 and rs2516837. Among these SNPs, rs3737787, rs2516841, rs2516839 and their haplotypes show significant association with T2DM in the Chinese population (14). Naukkarinen and colleagues indicates that the SNP rs2037658 is associated with USF1 level in tissues and the expression of the *APOE, ABCA1* and *AGT* genes. Consequently, it can influence the regulation of traits such as blood lipid, blood glucose, and blood pressure (15). Nonetheless, some research shows linkage disequilibrium between rs2073658 and rs3737787, suggesting that the rs3737787 polymorphism may actually lead to the alteration of lipid and glucose metabolism. Thus, with the information aforementioned, it can be rationally hypothesized that *USF1* gene is a susceptibility gene of T2DM related glucose and lipid metabolic abnormalities. To test this hypothesis, we tested the association between the polymorphism of rs3737787 in *USF1* gene and T2DM and its related glucose and lipid metabolic traits in the Beijing Fangshan Family Study.

## METHODS

### Study site and population

This study was conducted in Fangshan District in suburban Southwest Beijing, China. The population in Fangshan District is stable and unlikely to migrate. In addition, the genetic background of the population tends to be unique and the living environments are similar among individuals. Thus, the population is Fangshan District is suitable for genetic epidemiological studies, and has been used as the study population by our research group for years (16–18). The First Hospital of Fangshan District is the central hospital in the study, and provides services to the urban and rural population. Thus, sufficient cases have been proposed to enroll for the study. This study ran in conjunction with the Fangshan/Family-based Ischemic Stroke Study in China (FISSIC) (17).

### Procedure

Eligible probands were identified using the following criteria:

1. were diagnosed with T2DM according to World Health Organization (WHO) criteria (19) and
2. were patients in Fangshan District First Hospital or lived in the communities served by the hospital.
3. There was no evidence of acute infection, trauma or other situation of stress.
4. They did not have mitochondrial mutation related diabetes which was detected using polymerase chain reaction with restriction fragment length polymorphism (PCR-RFLP) to detect the tRNA 3243A/G mutation.
5. They did not have maturity onset diabetes of the young (MODY) which was determined by pedigree analysis and age of onset of diabetes.

Eligible families were chosen from the families of eligible probands using the following explicit inclusion criteria:

1. had at least one affected sibling and one living parent.
2. All the parents and the siblings agreed to participate in the study with informed consent and
3. did not have severe disabilities that affected their ability to participate in the study.

Families that had lived in distant regions and families without informed consent were excluded from the study.

After a family was enrolled, a questionnaire was administered by trained interviewers to each member of the family to obtain information on demographic characteristics, previous medical history, family history, and life habits (including cigarette smoking, alcohol consumption, physical activities, and diet). Physical examination data including height, weight, waist, and hip circumference were recorded by trained doctors using standardized processes. Blood samples were obtained via venipuncture by skilled nurses. DNA was extracted using phenol-chloroform approach, according to the standard protocol (20).

### Genotyping

For the rs3737787 polymorphism, the sequences of the forward and reverse primers used in the polymerase chain reaction are 5′-GGCCTGCAGTGGTGTGAAA-3′ and 5′-TCCAGTATCCAGCATGGAGACA-3′ (synthesized by Shanghai GeneCore BioTechnologies Co. Ltd.). Genomic DNA (100ng) is added to a buffer with deoxyribonucleoside triphosphates (10 mM × 0.25 μL), forward and reverse primers (25 μM × 0.25 μL, respectively), and *Taq* polymerase (2 units/μL × 0.5 μL) in a final volume of 25μL. After the reaction mixture is preincubated for 5 minutes at 94°C, 35 rounds of amplification were performed, consisting of denaturation at 94°C for 30 seconds, annealing at 54°C for 30 seconds, extension at 72°C for 30 seconds, and a final extension at 72°C for 5 minutes. Polymerase chain reaction products are subjected to digestion by *HpyCH4*IV overnight at 37°C. The products are electrophoresed on a 2.5% agarose gel (120V for 1 hour) with ethidium bromide staining and visualized under ultraviolet light (UVP, Cambridge, UK). Homozygous wild-type (CC) individuals show 96- and 33-base pair (bp) fragment, while heterozygous individual show three bands at 129, 96, and 33 bp, respectively. Homozygous rare-allele individuals, which cannot be digested by *HpyCH4*IV, show only 129-bp band.

### Statistical analyses

Most of the statistical analyses were conducted using SPSS software (SPSS for Windows, version 15.0; SPSS, Chicago, IL, USA) unless it was necessary to use other specified software. A *p* value less than 0.05 was considered statistically significant (two-tailed).

### Family-based linkage and association analyses

We used SIB-PAIR 1.00b to perform Mendelian error checking and genotype imputation in the family data and MERLIN 1.1.2 to perform variance component linkage analyses for blood lipid traits. The standard non-parametric linkage analysis used by MERLIN employs the Kong and Cox linear model to evaluate the evidence for linkage. This model is designed to discover small increases in allele sharing spread across a large amount of families, which is usually expected in a complex disease such as T2DM. The method of variance component first used for quantitative trait loci (QTL) mapping of complex diseases in the 1990s, and has become a common method of QTL linkage analysis (21). This method is to model the influence of alleles to traits to test the association, and, meanwhile, to model the covariance structure to test the linkage.

With SAS macro program, we used the family based association test (FBAT) method to test for the association between the SNP and the traits under additive genetic model. The FBAT method was developed by Rabinowitz and Laird based on transmission/disequilibrium test (TDT), which adopted score test to compute the statistic and then to conduct the statistical inference. While conserving the characteristic of TDT that avoids the effects of population stratification, FBAT also is suitable for various types of pedigrees and genetic models, thus, it is a popular method in genetic association studies.

We also use generalized estimating equations (GEE) to assess the relationship between the polymorphism and the related traits. GEE is a unification of two major approaches for linkage analysis of quantitative traits: variance components and Haseman-Elston regression. This method considers environmental and other covariates while evaluating the relationship (22).

## RESULTS

A total of 287 families, involving 812 individuals were included in this study, among which 796 individuals were genotyped successfully, giving a genotype success rate of 98.3%. The genotype frequencies of CC, CT, and TT were 59.3%, 34.7%, and 6.0%, respectively. There were no statistically significant differences in the distribution of genotypes between genders (Table 1). The allele frequencies of C and T were 0.769 and 0.231, respectively. The SNP is in Hardy-Weinberg equilibrium (*p*=0.175) (Table 2).

**Table 1:** The genotype distribution of rs3737787 of USF1 gene in deferent genders

Genotype	Male N (%)	Female N (%)	Total N (%)	χ^2^	*p*

CC	222(62.4)	250(56.8)	472(59.3)	3.484	0.175
CT	111(31.2)	165(37.5)	276(34.7)		
TT	23 (6.5)	25(5.17)	48(6.0)		
Total	356(100.0)	440(100.0)	796(100.0)		

**Table 2:** Hardy-Weinberg equilibrium test of rs3737787 of USF1 gene

Genotype	Observed count	Expected count	Genotype frequency	Allele	Observed count	Genotype frequency	χ^2^	*p*

CC	476	470.53	0.598	C	1224	0.769	1.189	0.276
CT	272	282.93	0.342					
TT	48	42. 53	0.060	T	368	0.231		
Total	796				1592			

### Non-parametric linkage analysis

Using MERLIN software, we analyzed the linkage between polymorphism of rs3737787 and T2DM and its related traits. We found that the polymorphism of rs3737787 may have a potential linkage relationship with the triglyceride and apolipoprotein E traits, with the logarithm of the odds (LODs) of 0.87 (*p*=0.02) and 1.96 (*p*=0.001), respectively. No other linkage relationships with quantitative traits were observed. We also detected a possible linkage between the polymorphism and T2DM related binary traits; however, all the LODs were less than 1.0, i.e. the potential linkage relationships were not statistically significant (Table 3).

**Table 3:** Linkage analysis of polymorphism of rs3737787 and T2DM related traits

Quantitative traits			Binary traits		
variable	LOD	*p*	variable	LOD	*p*

Waist circumference (cm)	0.00	0.50	T2DM	−0.41	0.90
Body mass index (kg/m^2^)	0.00	0.50	Metabolic syndrome	−0.60	0.90
Systolic blood pressure (mmHg)	0.00	0.50	Obesity	−0.77	1.00
Diastolic blood pressure (mmHg)	0.36	0.10	Hypertension	−0.26	0.90
Triglyceride (mmol/L)	0.87	0.02^a^	Hypertriglyceridemia	−0.54	0.90
Total cholesterol (mmol/L)	0.00	0.50	Hypercholesterolemia	−0.21	0.80
HDL-cholesterol (mmol/L)	0.00	0.50	Hyper-LDL-cholesterolemia	−0.43	0.90
LDL-cholesterol (mmol/L)	0.00	0.50	Hypo-HDL-cholesterolemia	0.13	0.20
Apolipoprotein A (g/L)	0.01	0.40	Dyslipidemia	−0.59	1.00
Apolipoprotein B (g/L)	0.08	0.30			
Apolipoprotein E (mg/dL)	1.96	0.001^a^			

a *p*<0.05

### Family based association study (FBAT)

Using FBAT method to analyze the relationship between polymorphism of rs3737787 and blood lipid levels in T2DM families, we found that C allele of rs3737787 was associated with levels of TG, TC, HDL-C, LDL-C, ApoB, and ApoE, and that, except for LDL-C and ApoE, all the indicators were associated with CC genotype, under the additive model (Table 4).

**Table 4:** FBAT analysis of polymorphism of rs3737787 and blood lipid traits under additive model

variable	genotype	fmy	S	E (S)	Var (S)	Z	*p*	allele	fmy	S	E (s)	Var (s)	Z	*p*

TG	CC	116	339.4	307.5	327.7	1.763	0.078	C	126	970.55	930.9	368.1	2.068	0.039^a^
	CT	118	291.75	315.9	334.5	−1.321	0.186	T	126	394.27	433.9	368.1	−2.068	0.039^a^
	TT	29	51.26	59	23.6	−1.598	0.110							
TC	CC	117	818.57	766.8	422.2	2.522	0.011^a^	C	127	2411.6	2342	558.9	2.943	0.003^a^
	CT	119	774.46	808.5	465.3	−1.579	0.114	T	127	1077.88	1147.4	558.9	−2.943	0.003^a^
	TT	29	151.71	169.5	89.9	−1.872	0.061							
HDL-C	CC	116	256.53	242	48	2.091	0.036^a^	C	126	757.06	738.6	66.1	2.274	0.023^a^
	CT	119	244	254.5	53.9	−1.43	0.152	T	126	353.66	372.2	66.1	−2.274	0.023^a^
	TT	28	54.83	58.8	12	−1.155	0.248							
LDL-C	CC	117	486.06	462.4	170.9	1.812	0.070^a^	C	127	1450.56	1416.7	232.1	2.225	0.026^a^
	CT	119	478.44	491.9	190	−0.979	0.328	T	127	665.72	699.6	232.1	−2.225	0.026^a^
	TT	29	93.64	103.8	40.1	−1.61	0.107							
APOA	CC	117	245.1	235.4	37.7	1.574	0.115	C	127	723.84	711.4	52.5	1.721	0.085
	CT	119	233.64	240.5	40.3	−1.083	0.279	T	127	339.78	352.2	52.5	−1.721	0.085
	TT	29	53.07	55.9	8.7	−0.951	0.342							
APOB	CC	117	167.62	159	18.5	2.014	0.044^a^	C	127	496.79	484.7	24.4	2.452	0.014^a^
	CT	119	161.55	166.8	20.4	−1.152	0.249	T	127	225.61	237.7	24.4	−2.452	0.014^a^
	TT	29	32.03	35.5	3.9	−1.74	0.082							
APOE	CC	102	608.5	570.5	489.7	1.715	0.087	C	111	1801.3	1743.7	656.1	2.249	0.024^a^
	CT	103	564.3	582.6	518	−0.804	0.422	T	110	764.3	821.9	656.1	−2.249	0.024^a^
	TT	26	100	119.7	97.3	−1.993	0.046^a^							

fmy, the number of informative families;

a *p*<0.05.

### Multi-factorial generalized estimate equation analysis (GEE)

Using the method of GEE to control the intra-familial correlation and to adjust for potential confounders (including age, gender, alcohol consumption, smoking status, labor, physical exercise, and life pressure), we found that the TT genotype of rs3737787 was statistically associated with triglyceride and apolipoprotein E (Table 5).

**Table 5:** GEE analysis of TT genotype of rs3737787 in USF1 gene and T2DM related traits^a^

variable	β	95%CI	*p*

Waist circumference (cm)	0.4	(0.6,1.4)	0.420
Body mass index (kg/m^2^)	0.4	(0.1,0.8)	0.091
Systolic blood pressure (mmHg)	1.1	(1.1,3.4)	0.314
Diastolic blood pressure (mmHg)	0.6–0.7	(−0.8,2.1)	0.369
Triglyceride (mmol/L)	−0.3	(−0.5,−0.1)	0.010^b^
Total cholesterol (mmol/L)	−0.1	(−0.2,0.1)	0.226
HDL-cholesterol (mmol/L)	0.0	(−0.0,0.1)	0.620
LDL-cholesterol (mmol/L)	0.0	(−0.1,0.1)	0.569
Apolipoprotein B (g/L)	−0.0	(−0.1,0.0)	0.424
Apolipoprotein E (mg/dL)	−0.4	(−0.7,−0.1)	0.007^b^

a The comparisons are between the CC and TT genotypes. All adjusted by age, gender, alcohol consumption, smoking status, labor, physical exercise, and life pressure;

b *p*<0.05.

## DISCUSSION

Many patients with type 2 diabetes mellitus die of atherosclerosis and its complications, while abnormalities in plasma lipoproteins and derangements in lipid metabolism rank as the most firmly established and best understood risk factors for atherosclerosis (23). The abnormal lipoprotein profile associated with insulin resistance, known as diabetic dyslipidemia, accounts for part of the elevated cardiovascular risk in patients with type 2 diabetes. Hypertriglyceridemia is the basic characteristic of lipid metabolism in T2DM individuals. Other lipid metabolic risks of atherosclerosis, such as low HDL-C, and high LDL-C, are highly related with hypertriglyceridemia (24). While diabetic patients often have LDL cholesterol levels near average, the LDL particles tend to be smaller and denser and thus more atherogenic. Other features of diabetic dyslipidemia include low HDL and elevated triglyceride levels. The Adult Treatment Panel III (ATP III) guidelines now recognize this cluster of risk factors and provide criteria for diagnosis of the “metabolic syndrome”. Moreover, the T2DM patients with dyslipidemia are more likely to suffer from some hepatic and biliary disorders such as nonalcoholic steatohepatitis (NASH) and biliary lithiasis (25, 26). Insulin resistance plays a vital role in dyslipidemia of T2DM individuals. Insulin resistance increases the blood VLDL and TG levels and reduces their clearance rates. A mechanism of the enhanced VLDL in T2DM patients is that excessive insulin can promote the activity of sterol regulatory element binding protein 1c (SREBP-1c), whose activation can promote the process of lipogenesis and lead lipid accumulate in liver. Consequently, the amount of triglyceride, the material of VLDL synthesis, is augmented (27). Apolipoprotein E is the ligand of both LDL receptor and LDL receptor-related protein. Apolipoprotein E plays an important role in removing triglyceride-rich lipoproteins (TRLs) remnant (28).

In this study, we first report the genotype frequency of rs3737787 of *USF1* gene in northern Chinese population. The results were in accordance with other studies in similar populations (29). In the linkage analysis, we found that the logarithm of the odds (LODs) for triglyceride and apolipoprotein E were statistically significant. This indicates that the rs3737787 polymorphism may either tend to transmit together with the putative pathogenic gene polymorphism of dyslipidemia in T2DM individuals, or be the pathogenic gene polymorphism per se. In the association study, we used the FBAT method to detect the association between the polymorphism and blood lipid profile. We have observed that the C allele of rs3737787 was associated with the levels of TG, TC, HDL-C, LDL-C, ApoB, and ApoE, and that, except for LDL-C and ApoE, all other indicators were associated with the CC genotype. However, after controlling the intra-familial correlation and adjusting for potential confounders, the only associated traits triglyceride and apolipoprotein E. These two traits are significantly associated with the TT genotype of rs3737787. Using different statistical approaches, we confirmed that the single nucleotide polymorphism rs3737787 is related to triglyceride and apolipoprotein E level in type 2 diabetes mellitus families. Association study and linkage analysis of candidate genes are two common approaches of identifying pathogenic genes. The results of the two supported each other, but have different emphases and advantages. Association studies mainly detect the association between disease and alleles in certain population, while linkage analysis usually focuses on whether the alleles are related to the transmission of certain diseases in pedigrees. While the former one emphasizes the gene frequency in population, the latter one underlines the genetic characteristics of genes. Our finding of a relationship between rs3737787 and triglyceride is in accordance with other research (30). However, the relationship between ApoE and rs3737787 has not been examined. This study is the first to suggest a potential relationship between the level of serum ApoE and the rs3737787 polymorphism of the *USF1* gene.

Usually, for complicated diseases such as T2DM or dyslipidemia, the associated polymorphisms do not lead to obvious defects in their translated proteins. They often tend to lie in the presumptive regulatory regions, such as promoters or enhancers in introns (14, 31). Minor changes in the level of expression of certain genes can produce pathological abnormalities. However, it has been suggested that complex metabolic diseases can be a consequence of small changes occurring in several genes in one metabolic pathway. There may be several causes of these: several individual changes in the regulatory regions of different genes in the same pathway or a change in a transcription factor common to the regulation of these genes. A study of Naukkarinen *et al*. found that carriers of USF1 risk allele (of the SNP rs2073658) show differential expression of downstream genes in fat biopsies (15). Three genes, ATP-binding cassette subfamily A (*ABCA1*), angiotensinogen (*AGT*), and apolipoprotein E (*APOE*), differed significantly in their expression between the two haplotype groups of USF1 (defined by SNPs in LD with rs3737787 and rs2073658). They also indicated that the most downregulated gene in the risk individuals was APOE, which is expressed in at risk individuals at only half of the levels of the non-risk ones. However, rs3737787 in the 3′-untranslated region (UTR) and rs2073658 in intron7 are in complete linkage disequilibrium (LD) (R^2^=0.93, D′=0.98). While we know that 3′-UTR can mediate the translational control of corresponding genes (32), it can be reasonably considered that the effects of different alleles of rs2073658 actually resulted from the LD relationship with rs3737787, i.e. rs3737787 may actually be the reason for the differences between the individuals of different risk. Furthermore, rs3737787 is located in the promoter region (−789) of junctional adhesion molecule-1 (*JAM*1, also known as adjacent platelet F11 receptor, *F11R*), which was first discovered to be a surface protein on human platelets, and has been found to be associated with central obesity and systolic blood pressure in the Chinese population (33). Considering these information, it is suggested that the association might also be partly due to causative variants in *JAM*1.

## CONCLUSION

In conclusion, our study shows a relationship between an important polymorphism of *USF1* gene and blood lipid profile in Chinese type 2 diabetes mellitus families. However, these findings need to be confirmed in other population and with larger sample sizes, together with a functional analysis of the gene with such polymorphism.
